# Role of laparoscopy in non-trauma emergency pediatric surgery: a 5-year, single center experience a retrospective descriptive study with literature review

**DOI:** 10.1186/1756-0500-5-550

**Published:** 2012-10-05

**Authors:** Tariq O Abbas, Ahmed Hayati, Mansour Ali

**Affiliations:** 1 Pediatric Surgery Department, Hamad General Hospital, Doha, 3050, Qatar

**Keywords:** Laparoscopy, Pediatric, Non-trauma, Emergency

## Abstract

**Background:**

Although laparoscopy is rapidly becoming the abdominal surgical modality of choice in adults, there are obstacles to its use in children. We analyzed our experience with pediatric laparoscopic surgery over the past 5 years, with particular emphasis on emergency procedures.

**Findings:**

We retrospectively evaluated the records of patients aged <14 years who had undergone laparoscopic procedures for non-trauma emergency conditions at our institution from January 2006 to December 2010. The clinical parameters evaluated included operation time, total length of hospital stay, and postoperative complications.

During the 5-year study period, 482 laparoscopic procedures were performed on patients aged <14 years, comprising 300 emergency and 182 elective operations. The majority of procedures were laparoscopic appendectomies, with most of the others being resections of ovarian cysts or Meckel’s diverticulae, or adhesiolyses. We observed an improvement in outcomes over the 5-year period, as shown by shorter operation times and shorter postoperative hospital stays. The numbers of laparoscopic procedures performed increased over time.

**Conclusions:**

Pediatric laparoscopic surgery for emergency conditions provides excellent results, including better exposure and cosmetic outcomes than laparotomy. At our institution, the numbers and types of laparoscopic procedures performed have increased over time, and the outcomes of laparoscopic procedures have improved.

## Findings

### Background

Laparoscopic surgery in children is not new, with pediatric surgeons being among the pioneers of laparoscopic surgery in the early 1970s. However, surgeons are only recently beginning to realize the great potential of this minimally invasive approach in children [1].

About 60% of abdominal operations in children can be performed laparoscopically. Although most types of laparoscopic surgery are not performed frequently, the feasibility of this technique for routine use is excellent [2]. With appropriate training and experience, minimal access surgery (MAS) can safely be used to treat children with a wide variety of conditions, with minimal morbidity and mortality [3].

We report here our experience with laparoscopic procedures performed for non-trauma emergency conditions in children at our institution over the past 5 years. We also describe the improvements that occurred over this time period, as shown by several measurable clinical indicators.

## Methods

We retrospectively evaluated the medical records of all patients aged <14 years who had undergone laparoscopic surgery at our institution for non-trauma-related conditions from January 2006 to December 2010. All patients underwent preoperative clinical examination and routine laboratory investigations.

Laparoscopic exploration was performed through a 5-mm (if the patient was aged <2 years) or 10-mm (if the patient was aged >2 years) supra-umbilical port, using closed techniques and a 5-mm camera. Secondary 3–5-mm ports were placed under direct vision as required.

Parameters assessed included the patient’s age at operation, radiological and laparoscopic findings, operative interventions, and postoperative outcomes. Markers of surgical outcome included operation time, length of hospital stay, postoperative complications, and duration of follow-up. Data were expressed as the mean ± standard error of the mean.

## Results

**Figure 1:**
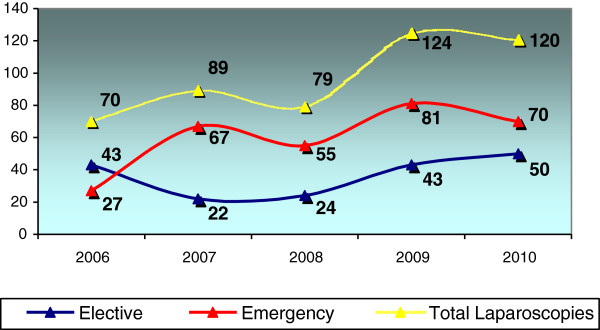
**Numbers of pediatric laparoscopic surgical procedures performed annually at our institution over the 5-year period from January 2006 to December 2010.** The proportions of emergency and elective procedures are shown.

Over the 5-year study period, 482 laparoscopic procedures were performed in patients aged <14 years, of which 300 were emergency procedures and 182 were elective procedures. The number of emergency laparoscopic procedures performed per year almost tripled from 2006 (27 procedures) to 2010 (70 procedures) (Figure 1). This increase may reflect an increase in patient numbers at our institution as well as an increase in the proportion of procedures performed laparoscopically. The number of elective laparoscopic procedures performed per year decreased during the first half of the study period, and then increased during the second half to return to numbers close to those observed at the beginning of the study period.

**Figure 2:**
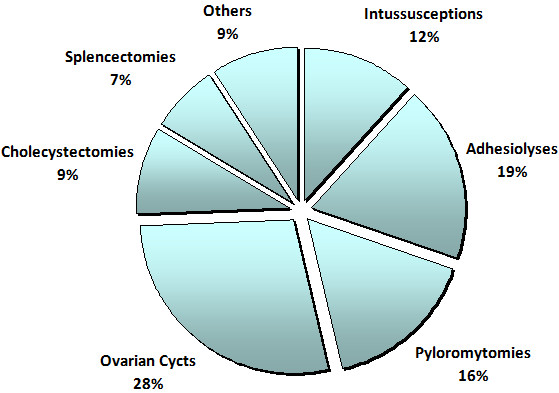
Proportions of types of emergency laparoscopic procedures performed over the 5-year study period.

Overall, 61.4% of all laparoscopic procedures performed during the study period were for emergency conditions. Of these emergency laparoscopic procedures, the majority (84.7%) were appendectomies, followed by neonatal ovarian cystectomies (Figure 2).

The mean patient age at surgery varied according to the type of procedure performed. For example, the mean age of patients undergoing appendectomy was 10.01 ± 2.69 years, and of patients undergoing ovarian cystectomy was 18.9 days. The mean operation time also varied according to the procedure performed, being 76.5 ± 35.8 min for appendectomy and 31 min for pyloromyotomy.

Complications occurred in 1.35% of patients who underwent appendectomy, compared with 0% of patients who underwent adhesiolysis.

The mean duration of hospital stay ranged from 2.75 days following cholecystectomy to 26 days following adhesiolysis (Table 1). Laparoscopic exploration changed the preoperative diagnosis in 10.6% of patients.

**Table 1:** Clinical and procedural parameters of pediatric patients who underwent different types of emergency laparoscopic surgery at our institution

**Procedure**	**Number of cases**	**Age at operation**	**Number (%) of Conversions to open surgery**	**Operation time (min): Mean ± SEM**	**Mean postoperative Hospital stay (days)**	**Number (%) of complications**
Appendectomy	221	10.0 ± 2.7 years	7 (3.7%)	75.5 ± 35.1	3.2 ± 1.2	3 (1.5%)
Ovarian Cystectomy	12	18.9 days (+ one patient 12 years)	0 (0%)	69.6 ± 23	4.75	0 (0%)
Adhesiolysis	8	1.7 months	2 (25%)	98.8	26.1	0 (0%)
Reduction of Intussusception	5	4 months	3 (60%)	66.0	3.0	0 (0%)
Pyloromyotomy	7	59.2 days	0 (0%)	31.0	4.6	1 (0.1%)
Cholecystectomy	4	11 years	0 (0%)	55.0	2.8	0 (0%)
Other	4	18.2 months	2 (50%)	81.3	23.5	0 (0%)

**Figure 3:**
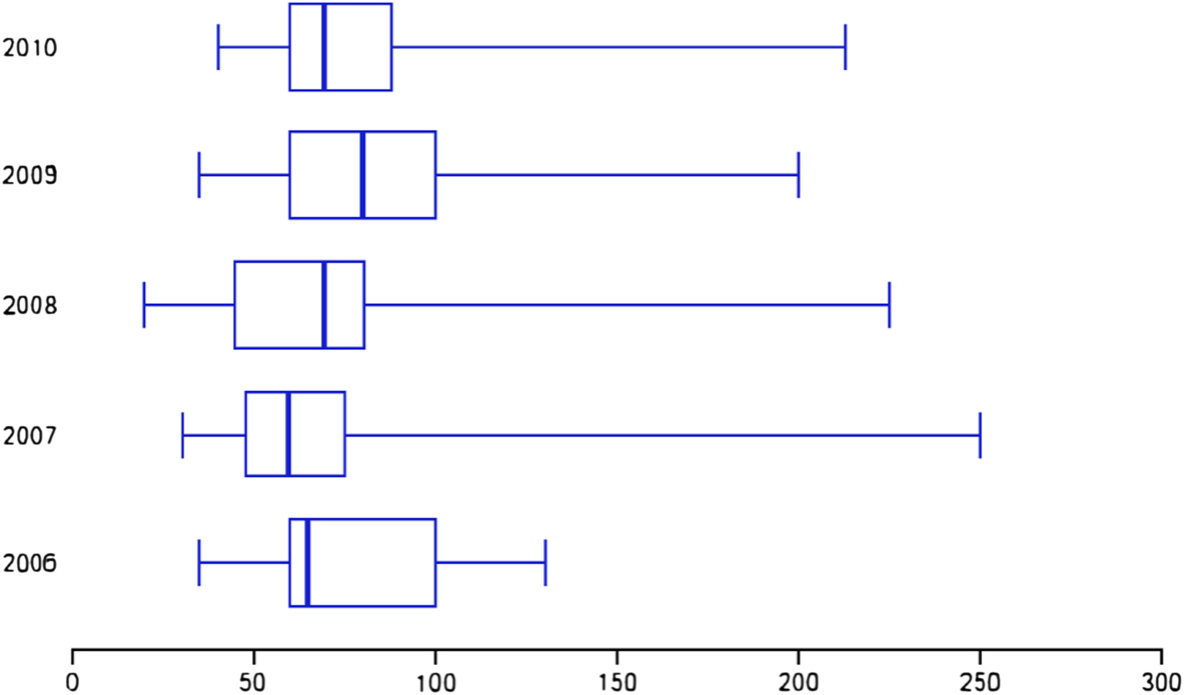
Box plots of operation time (min) over the 5-year study period.

**Figure 4:**
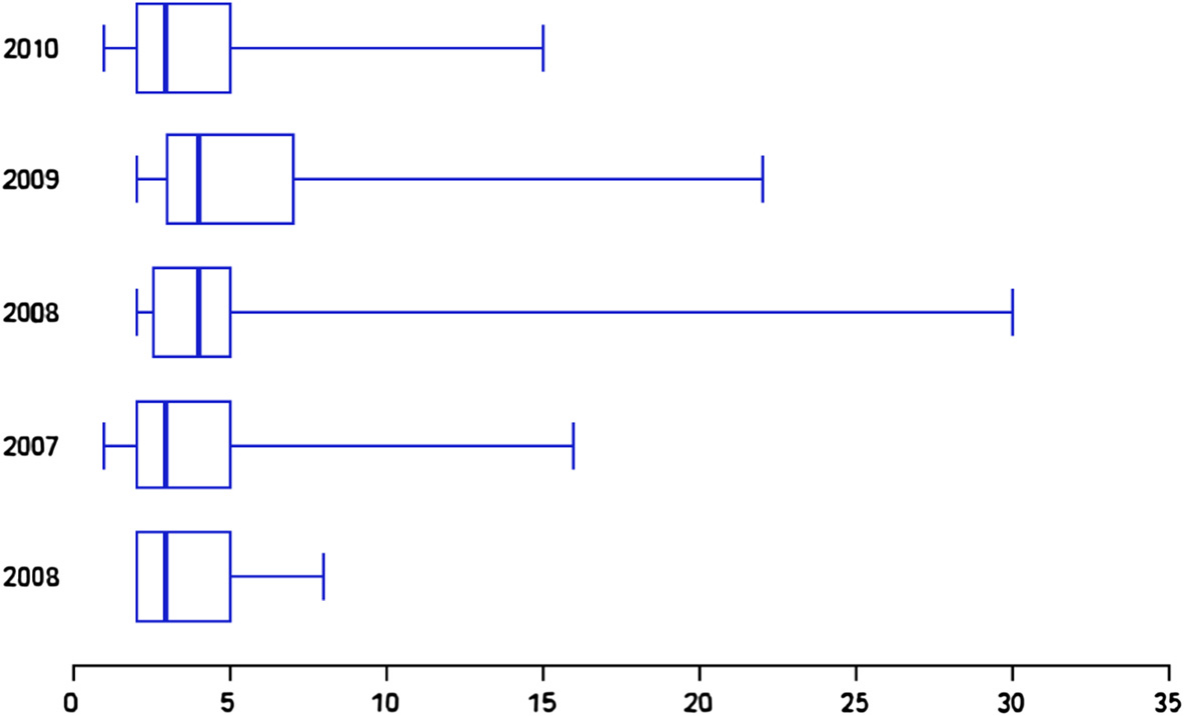
Box plots of postoperative hospital stay (days) over the 5-year study period.

Investigation of perioperative clinical parameters showed that operation time (Figure 3) and total length of hospital stay (Figure 4) tended to decrease during the study period.

## Discussion

Over the past two decades, laparoscopy has been performed in pediatric patients primarily for diagnostic purposes. In the early 1990s, there was a rapid expansion in laparoscopic surgery for adults, due to the success of laparoscopic cholecystectomy. However, interest in laparoscopic surgery in children remained confined to a few enthusiasts, while most pediatric surgeons adopted a “wait and see” attitude. More recently, increased surgical experience and advances in miniaturized instrumentation have resulted in increased acceptance of pediatric laparoscopic procedures [1].

Minimal-access surgery (MAS) is rapidly becoming the approach of choice for a variety of surgical procedures in adults, but its use in children remains a relative novelty. Most pediatric surgeons have ongoing concerns about the morbidity of this modality, due to the cumbersome nature of the instruments and the technical difficulties associated with two-dimensional views [3].

MAS has several advantages over open surgery, including smaller incisions, shorter hospital stays, and a more rapid return to preoperative activities, making it more appealing to patients [4].

Recent advances in minimally invasive surgery have clearly benefited children with abdominal pain who require surgery. As techniques improve and instruments become smaller, the use of these methods is expected to increase [5]. Technological advances, combined with increasing surgical and anesthetic expertise, have widened the range of laparoscopic procedures currently performed in children [6]. The most frequently performed emergency procedures are described below.

Laparoscopic *appendectomy* has resulted in a shorter median length of stay, a trend towards fewer postoperative infectious complications, and fewer clinic visits than after open appendectomy, indicating that it is a safe and effective procedure for the treatment of patients with perforated appendicitis [7]. We performed 221 laparoscopic appendectomies, accounting for 84.7% of all the pediatric appendectomies performed at our institution during the study period. Our complication rate was 1.35%. Conversion to open appendectomy was required in 3.67% of cases.

Laparoscopically assisted *small bowel* surgery allows the surgeon to perform the dissection laparoscopically and then deliver the loops of intestine through a small incision, permitting anastomoses to be performed outside the abdominal cavity [8]. In two of our patients, however, laparoscopic *pyloromyotomy* was unsuccessful, resulting in the need for revision pyloromyotomy. This may have been due to the small number of laparoscopic pyloromyotomies performed during the study period, and the associated learning curve.

A laparoscopic approach may be used to manage *intussusception*. Although some patients will require conversion to an open procedure, the laparoscopic approach should be considered safe and effective when attempted radiological reduction is unsuccessful [9]. During the 5-year study period, we performed only five laparoscopic reductions of intussusception, with no complications. However, the remaining patients underwent laparotomy.

Laparoscopic *splenectomy* permits removal of the spleen without the morbidity associated with a large incision. Most surgeons perform this procedure using three small access ports and one larger port through which the spleen is eventually removed [4]. A previous study reported that >85% of laparoscopic splenectomies were performed to treat hematologic disorders [10]. All three of the laparoscopic splenectomies performed at our institution during the 5-year study period were for hematologic disorders.

When performed by an experienced operator, laparoscopic *ovarian cystectomy* is a safe and beneficial alternative to laparotomy in children [11]. We performed 12 laparoscopic ovarian cystectomies, with a mean patient age at operation of 18.9 days. Although most of these cysts were simple neonatal cysts, surgery was required because they were >4 cm in size or because ultrasonography showed features indicating a high likelihood of torsion.

In one study, the overall complication rate of MAS was reported to be 4% (26 of 626 procedures), with no deaths [3]. Our results were similar, with a complication rate of 1.33% (4 of 300 patients) and no deaths.

The infection rate among our patients was 1.33%. Interestingly, it has been found that in patients with peritonitis, pathogenic organisms such as *Escherichia coli* use different metabolic pathways after laparoscopy than after laparotomy [12].

In general, laparoscopy results in a shorter hospital stay, reduced potential for complications such as wound infection, and better cosmetic results. Laparoscopic exploration in emergency situations permits the surgeon to plan appropriate port placement depending on findings. Laparoscopic exploration changed the preoperative diagnosis in 10.6% of our patients, suggesting that laparoscopy provides better exposure for exploration in emergency cases than laparotomy, which is particularly useful when the diagnosis in not clear preoperatively. However, the acquisition of laparoscopic equipment and performance of laparoscopic procedures are associated with increased financial costs.

## Conclusion

In conclusion, laparoscopic surgery has excellent results, with better exposure and cosmetic outcomes than laparotomy, in children requiring emergency surgical intervention. At our institution, the types and numbers of pediatric emergency laparoscopic procedures performed have increased over time.

### Availability of Supporting Data

The data sets supporting the findings are included within the article and its additional files.

## Abbreviations

(MAS): Minimal access surgery.

## Competing interests

The authors declare that they have no competing interests.

## Authors’ contributions

TA helped to design and coordinate the study, participated in data collection, and drafted the manuscript. AH participated in data collection. MA conceived the study, and helped to design and coordinate it. All authors read and approved the final manuscript.

## References

[B1] Tam PKH Laparoscopic surgery in children Arch Dis Child 2000 82 240 243 10.1136/adc.82.3.240 10685931 PMC1718250

[B2] Ure BM Bax NM van der Zee SC Laparoscopy in infants and children: A prospective study on feasibility and the impact on routine surgery J Pediatr Surg 2000 35 1170 1173 10.1053/jpsu.2000.8720 10945688

[B3] Chen MK Schropp KP Lobe TE Complications of minimal-access surgery in children J Pediatr Surg 2006 31 1161 1165 8863256 10.1016/s0022-3468(96)90109-8

[B4] Zitsman JL Pediatric minimal-access surgery: update Pediatrics 2006 118 304 308 10.1542/peds.2005-2736 16818579

[B5] Mattei P Minimally invasive surgery in the diagnosis and treatment of abdominal pain in children Curr Opin Pediatr 2010 19 338 343 17505197 10.1097/MOP.0b013e32810c8eaf

[B6] Wells J Jawaheer G Laparoscopy in children 2010 28 27 32

[B7] Taqi E Al Hadher S Ryckman J Su W Aspirot A Puligandla P Flageole H Laberge JM Outcome of laparoscopic appendectomy for perforated appendicitis in children J Pediatr Surg 2008 43 893 895 10.1016/j.jpedsurg.2007.12.033 18485961

[B8] Diamond IR Langer JC Laparoscopic-assisted versus open ileocolic resection for adolescent Crohn disease J Pediatr Gastroenterol Nutr 2001 33 543 547 10.1097/00005176-200111000-00006 11740226

[B9] Kia KF Mony VK Drongowski RA Golladay ES Geiger JD Hirschl RB Teitelbaum DH Laparoscopic vs open surgical approach for intussusception requiring operative intervention J Pediatr Surg 2005 40 281 284 10.1016/j.jpedsurg.2004.09.026 15868598

[B10] Reddy VS Phan HH O’Neill JA Neblett WW Pietsch JB Morgan WM Cywes R Laparoscopic versus open splenectomy in the pediatric population: a contemporary single- center experience Am Surg 2001 67 859 863 discussion 863–864 11565764

[B11] Danzer E Schier F Gorsler C Pediatric Endosurgery & Innovative Techniques 2001 5 349 353 10.1089/10926410152776315

[B12] Didema B Hüseyinb B Osman Y Yasemin B Necati G Canan T Early effects of laparotomy and laparoscopy on bacterial behavior and proinflammatory cytokines on bacterial peritonitis in rats I: Escherichia coli J Pediatr Surg 2008 43 1494 1501 10.1016/j.jpedsurg.2008.01.004 18675641

